# Supplementary material for: The Origin of the Non-Constancy of the Bulk Resistance of Ion-Selective Electrode Membranes within the Nernstian Response Range
Source: Membranes (Basel). 2021 May 7;11(5):344. doi: 10.3390/membranes11050344 (PMC8150337; doi:10.3390/membranes11050344)
Supplement: Supplementary file 1 [file membranes-11-00344-s001.zip › membranes-1203474-supplementary.pdf]

membranes

Supplementary Information

The Origin of the Non-Constancy of the Bulk Resistance of Ion-Selective Electrode Membranes within the Nernstian Response Range

Valentina Keresten, Elena Solovyeva and Konstantin Mikhelson *

Chemistry Institute, c/o St.Petersburg State University, 26 Universitetsky Prospect, Stary Peterhof 198504, St. Petersburg, Russia; v_lukina@list.ru (V.M.K.); solovyeva.elena.v@gmail.com (E.V.S.)
* Correspondence: konst@km3241.spb.edu

SUMMARY

Number of pages: 4
Number of Figures: 3
Number of Tables: 3

Citation: Keresten, V.; Solovyeva, E.; Mikhelson, K. The Origin of the Non-Constancy of the Bulk Resistance of Ion-Selective Electrode Membranes within the NernstianResponse Range. *Membranes* **2021**, *11*, 344. https://doi.org/10.3390/membranes11050344

Academic Editor: Beata Paczosa-Bator

Received: 14 April 2021
Accepted: 4 May 2021
Published: 7 May 2021

Publisher's Note: MDPI stays neutral with regard to jurisdictional claims in published maps and institutional affiliations.

Copyright: © 2021 by the authors. Licensee MDPI, Basel, Switzerland. This article is an open access article distributed under the terms and conditions of the Creative Commons Attribution (CC BY) license (http://creativecommons.org/licenses/by/4.0/).

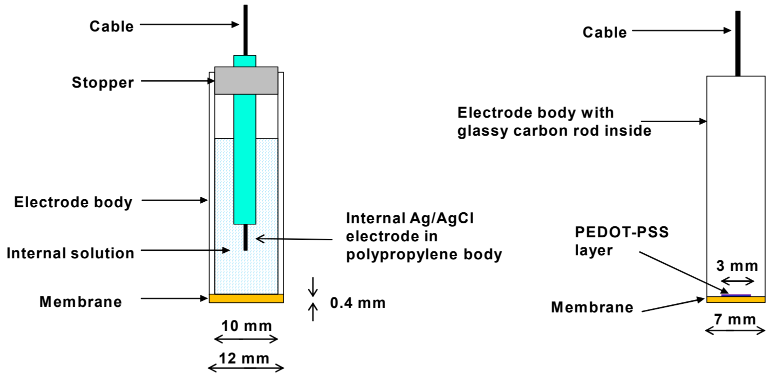

Figure S1. Electrode constructs. Left: classical Ion-Selective Electrode (ISE) with internal reference solution and internal reference electrode. Right: solid-contact electrode.

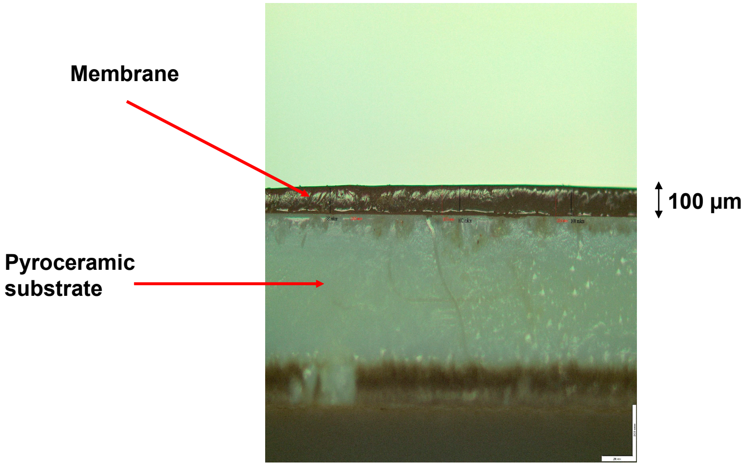

Figure S2. Cross-section of the membrane with thickness of 100 μm on a pyroceramic substrate.

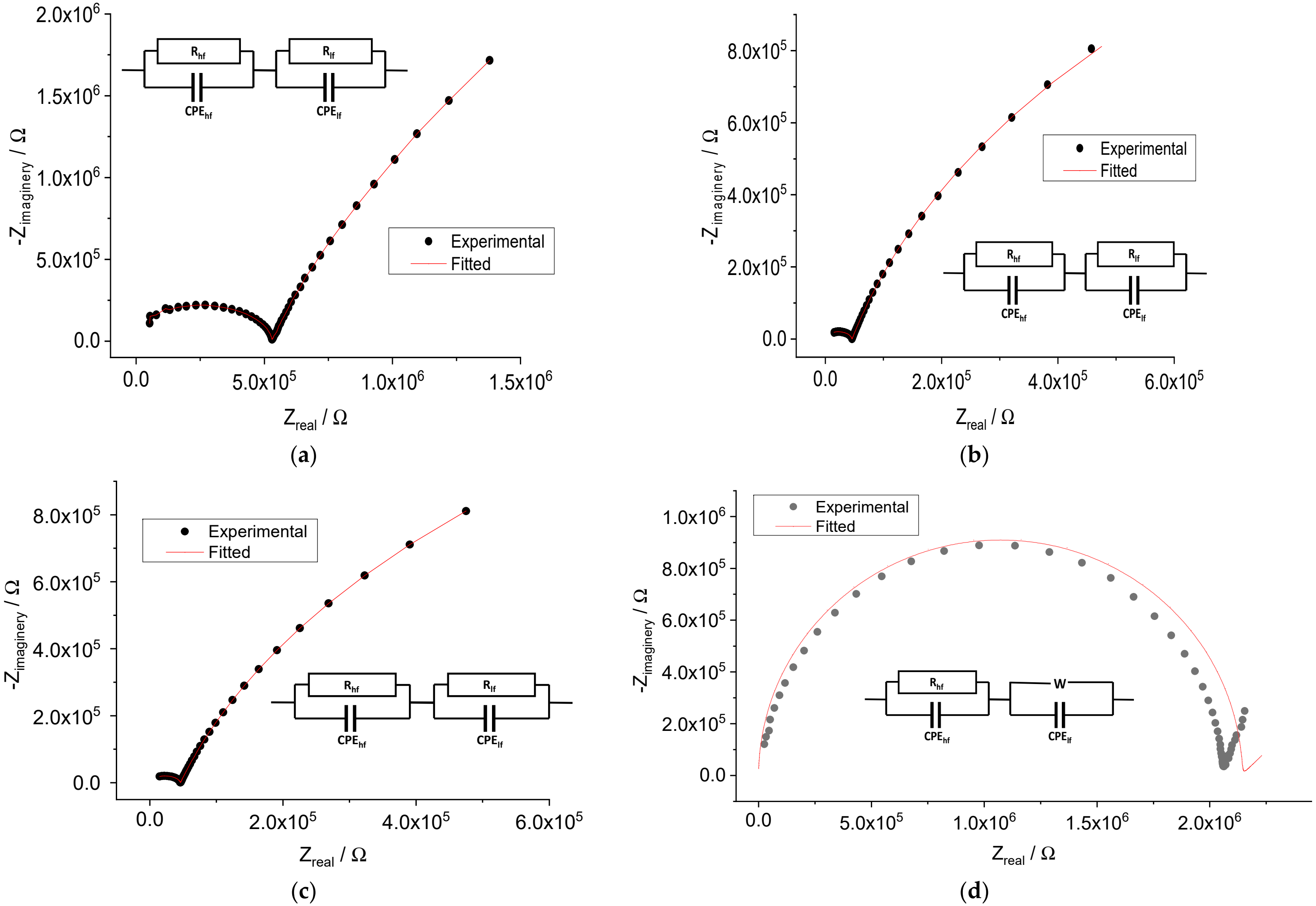

Figure S3. Nyquist plots of the impedance spectra of dry membranes. (**a**) K-; (**b**) Ca; (**c**) Cd; (**d**) NO_3.

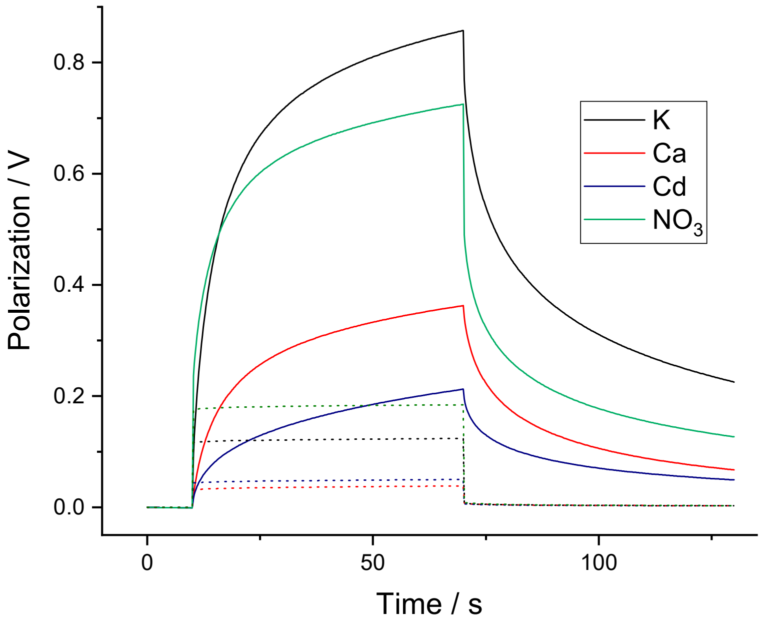

Figure S4. Chronopotentiometric curves (“slow” protocol) obtained by passing current with density of 1.27·10^{-7} A/cm^2 across dry membranes (solid lines) and membranes equilibrated with Solution #10 (dotted lines).

Table S1. Ohmic drops (V) registered in “fast” and “slow” chronopotentiometric measurements, and S/F: the ratio of the value obtained in “slow” protocol over that obtained in “fast” protocol.

**logTCI**	**logIS**	**K-ISE**			**Ca-ISE**			**Cd-ISE**			**NO_3-ISE**		
		**Fast**	**Slow**	**S/F**	**Fast**	**Slow**	**S/F**	**Fast**	**Slow**	**S/F**	**Fast**	**Slow**	**S/F**
−1.02	−1.28	0.107	0.109	1.02	0.019	0.019	1.02	0.025	0.026	1.02	0.180	0.180	1.01
−1.28	−1.46	0.134	0.138	1.03	0.029	0.030	1.03	0.028	0.028	1.03	0.173	0.177	1.03
−1.46	−1.47	0.108	0.110	1.02	0.020	0.020	1.00	0.025	0.026	1.03	0.175	0.177	1.02
−1.78	−1.88	0.107	0.109	1.02	0.019	0.019	1.02	0.026	0.026	1.02	0.180	0.185	1.03
−1.79	−1.79	0.115	0.117	1.02	0.025	0.025	1.01	0.025	0.026	1.03	0.155	0.159	1.02
−2.27	−2.37	0.098	0.101	1.03	0.017	0.018	1.03	0.024	0.025	1.02	0.156	0.160	1.03
−2.65	−2.94	0.109	0.111	1.02	0.020	0.020	1.03	0.028	0.029	1.02	0.152	0.156	1.02
−2.75	−2.86	0.113	0.113	1.01	0.021	0.022	1.02	0.028	0.029	1.02	0.155	0.157	1.01
−2.82	−3.03	0.117	0.120	1.02	0.021	0.022	1.02	0.031	0.031	1.01	0.163	0.164	1.00
−3.57	−3.82	0.113	0.116	1.03	0.030	0.031	1.03	0.043	0.044	1.02	0.170	0.176	1.03
−4.15	−4.30	0.168	0.172	1.02	0.067	0.069	1.02	0.083	0.086	1.03	0.255	0.259	1.01
Dry membranes		0.047	0.058	0.059	1.03	0.053	0.054	1.03	0.006	0.006	1.03	0.125	1.02

Table S2. Resistivity of the ISE membranes (MΩ·m) obtained by chronopotentiometric (fast protocol) and impedance measurements.

log(TCI)	log(IS)	K-ISE		Ca-ISE		Cd-ISE		NO_3-ISE	
		Chrono-potentio-metry	Impedance	Chrono-potentio-metry	Impedance	Chrono-potentio-metry	Impedance	Chrono-potentio-metry	Impedance
−1.02	−1.28	0.171	0.166	0.025	0.025	0.055	0.054	0.395	0.391
−1.28	−1.46	0.214	0.213	0.038	0.037	0.060	0.059	0.380	0.377
−1.46	−1.47	0.172	0.169	0.026	0.024	0.055	0.053	0.384	0.380
−1.78	−1.88	0.171	0.171	0.025	0.024	0.056	0.055	0.396	0.388
−1.79	−1.79	0.184	0.180	0.033	0.032	0.054	0.053	0.341	0.336
−2.27	−2.37	0.157	0.150	0.023	0.022	0.052	0.050	0.343	0.331
−2.65	−2.94	0.174	0.169	0.026	0.025	0.061	0.060	0.335	0.329
−2.75	−2.86	0.180	0.178	0.028	0.027	0.061	0.060	0.341	0.335
−2.82	−3.03	0.187	0.183	0.028	0.027	0.067	0.065	0.359	0.349
−3.57	−3.82	0.181	0.178	0.040	0.039	0.093	0.092	0.375	0.372
−4.15	−4.30	0.268	0.266	0.089	0.083	0.180	0.169	0.562	0.536
Dry membrane		0.092	0.090	0.070	0.069	0.012	0.012	0.274	0.273

Table S3. Water uptake (weight %) by the membranes equilibrated with mixed solutions.

log(TCI)	K-membrane		Ca-membrane		Cd-membrane		NO_3-membrane	
	Mean	SD	Mean	SD	Mean	SD	Mean	SD
−1.02	0.32	0.30	2.59	0.36	4.05	0.24	0.40	0.27
−1.28	0.33	0.26	2.55	0.25	4.18	0.35	0.36	0.19
−1.46	0.34	0.17	2.58	0.36	4.07	0.34	0.40	0.20
−1.78	0.34	0.17	2.60	0.26	4.05	0.37	0.48	0.22
−1.79	0.34	0.27	2.67	0.27	4.26	0.35	0.57	0.28
−2.27	0.37	0.19	2.51	0.27	4.39	0.36	0.33	0.31
−2.65	0.40	0.30	2.81	0.24	4.36	0.33	0.36	0.39
−2.75	0.40	0.20	2.81	0.28	4.37	0.36	0.41	0.32
−2.82	0.42	0.21	2.80	0.29	4.47	0.37	0.59	0.17
−3.57	0.50	0.36	3.04	0.31	4.80	0.37	1.00	0.24
−4.15	0.59	0.29	3.42	0.24	5.35	0.44	1.71	0.42
